# ﻿New studies on *Apiospora* (Amphisphaeriales, Apiosporaceae): epitypification of *Sphaeriaapiospora*, proposal of *Ap.marianiae* sp. nov. and description of the asexual morph of *Ap.sichuanensis*

**DOI:** 10.3897/mycokeys.92.87593

**Published:** 2022-08-23

**Authors:** Ángel Pintos, Pablo Alvarado

**Affiliations:** 1 Interdisciplinary Ecology Group, Universitat de les Illes Balears, Ctra. Valldemossa Km 7,5, 07122 Palma de Mallorca, Spain Universitat de les Illes Balears Palma de Mallorca Spain; 2 ALVALAB, Dr. Fernando Bongera st., Severo Ochoa bldg. S1.04, 33006 Oviedo, Spain ALVALAB Oviedo Spain

**Keywords:** *Apiosporaceae*, *Ascomycota*, *Sordariomycetes*

## Abstract

In the present work, an epitype for *Sphaeriaapiospora*, the basionym of the type species of the genus *Apiospora*, *Apiosporamontagnei*, is selected among collections growing in the host plant species reported in the original protologue, *Arundomicrantha*. Most samples obtained from localities near that of the lectotype (Perpignan, France) belong to the same species, which is not significantly different from the clade previously named *Ap.phragmitis*, suggesting that this name is a later synonym of *Ap.montagnei*. In addition, the name *Ap.marianiae* is here proposed to accommodate a newly discovered species found in the Balearic Islands (Spain), and the asexual state of *Ap.sichuanensis* is described for the first time from samples growing in the same islands.

## ﻿Introduction

*Apiospora* Sacc. is the type genus of family *Apiosporaceae* K.D. Hyde, J. Fröhl., Joanne E. Taylor & M.E. Barr. It occurs worldwide, and includes important pathogens and saprophytes of animals, plants and seaweeds (Heo et al. 2018, Park et al. 2018, Wang et al. 2018, Kwon et al. 2021). Genus *Apiospora* was built around *Apiosporamontagnei* Sacc. (Saccardo 1875), a replacement name for *Sphaeriaapiospora* Durieu & Mont. (Bory de Saint-Vincent and Durieu de Maisonneuve 1849). For a long time, *Apiospora* was considered a sexual state of genus *Arthrinium* Kunze, and both were even formally synonymyzed by Crous and Groenewald (2013), but recently shown to represent independent clades and separated again by Pintos and Alvarado (2021). These authors concluded that although the morphology of the original collections of *S.apiospora* (≡ *Ap.montagnei*) does not allow to link them with a unique phylogenetic clade, they should nest inside the clade containing most other species with basauxically-generated rounded/lenticular conidia that occur mainly on Poaceae (but also many other plant families, seaweeds and animals) worldwide (including tropical and subtropical regions), and differ from species in the clade of *Arthrinium*, which have variously shaped conidia, a narrower host range (mainly, but not exclusively, Cyperaceae and Juncaceae), and occur in temperate, cold or alpine (but not tropical or subtropical) regions. This way, Pintos and Alvarado (2021) selected a lectotype for *S.apiospora* (≡ *Ap.montagnei*), and fixed the phylogenetic limits of *Apiospora*, proposing the necessary combinations at species rank. Later authors followed this approach (Crous et al. 2021; Tian et al. 2021), and genomic analyses seem to confirm their taxonomic decision (Sørensen et al. 2022). A third group of species formerly placed within *Arthrinium*, including *Ar.urticae* M.B. Ellis (Ellis 1965) and *Ar.trachycarpi* C.M. Tian & H. Yan (Yan et al. 2019), are probably unrelated to *Arthrinium* or *Apiospora* (Tang et al. 2021), and therefore deserve to be classified in a different genus.

Despite these important taxonomic changes, the exact identity of the type species of *Apiospora*, *Ap.montagnei*, still remains uncertain. Pintos and Alvarado (2021) discussed the host plants mentioned by Bory de Saint-Vincent and Durieu de Maisonneuve (1849), concluding that the lectotype (collected near Perpignan, France) was found on *Arundomicrantha* or *Aru.donaciformis*. Only four species of *Apiospora*, *Ap.iberica* (Pintos & P. Alvarado) Pintos & P. Alvarado, *Ap.italica* (Pintos & P. Alvarado) Pintos & P. Alvarado, *Ap.marii* (Larrondo & Calvo) Pintos & P. Alvarado and *Ap.phragmitis* (Crous) Pintos & P. Alvarado had been recorded in *Arundo* spp. (Pintos et al. 2019, Pintos and Alvarado 2021), but since *Ap.iberica* and *Ap.italica* are relatively rare, *Ap.marii* and *Ap.phragmitis* were considered the most probable synonyms of *Ap.montagnei* (Pintos and Alvarado 2021).

In the present work, several collections of *Apiospora* growing on Arundoaff.micrantha in northeastern Spain and the Balearic Islands were analyzed, and an epitype of *Ap.montagnei* selected among them to fix the identity of this species. In addition, a newly discovered species found in the same region is described and given a new name, and the asexual state of *Ap.sichuanensis* Samarak., Jian K. Liu & K.D. Hyde is described for the first time.

## ﻿Materials and methods

### ﻿Isolates

Methods employed to isolate the sexual and asexual states are described in Pintos and Alvarado (2021). The samples were deposited in the fungarium of the Muséum National d´Histoire Naturelle (**PC**; Paris, France) and the Fungarium of the University of Vienna (**WU**; Vienna, Austria). Living cultures were deposited in Fungal collection at the Westerdijk Fungal Biodiversity Institute (**CBS**; Utrecht, The Netherlands).

### ﻿Morphology

Samples were studied with a Zeiss Axioscope compound microscope operating with differential interference contrast (DIC). Images were obtained with a FLIR camera using open source software Microscopia Oberta (A. Coloma). Measurements were taken with FIJI win64 ImajeJ software, and reported as follows: maximum value in parentheses, range between the mean plus and minus the standard deviation, minimum value in parentheses, and the number of elements measured in parentheses. For some images of conidiophores, the image stacking software Zerene Stacker v. 1.04 (Zerene Systems LLC, Richland, WA, USA) was employed. Morphological descriptions were based on fertile cultures growing on 2% MEA (20 g/L malt extract, 20 g/L soy peptone, 15 g/L agar, pH 7) at room temperature.

### ﻿Phylogenetic analysis

Total DNA was extracted from cultured isolates and dried fungarium specimens employing a modified protocol based on Murray and Thompson (1980). Amplification reactions (Mullis and Faloona 1987) included 35 cycles with an annealing temperature of 54 °C. Primers ITS1F and ITS4 (White et al. 1990, Gardes and Bruns 1993) were employed to amplify the ITS1-5.8S-ITS2 nrDNA region (ITS), while LR0R and LR5 (Vilgalys and Hester 1990, Cubeta et al. 1991) were used for the 28S nrDNA region (LSU), EF1-728F, EF1-983F and EF1-1567R (Carbone and Kohn 1999, Rehner and Buckley 2005) for the translation elongation factor 1 alpha (tef1) gene, and T1, Bt2a, and Bt2b (Glass and Donaldson 1995; O’Donnell and Cigelnik 1997) for the β-tubulin gene (tub2). PCR products were checked in 1% agarose gels, and positive reactions were sequenced with one or both PCR primers. Chromatograms were checked searching for putative reading errors in MEGA v. 5.0 (Tamura et al. 2011), and these were corrected.

A single alignment was made using: 1) ITS1-5.8S-ITS2 nrDNA, 2) 28S nrDNA, 3) tef1 region between 3’ extreme of intron 1 and the 5’ extreme of the exon between introns 2 and 3, and 4) tub2 region between intron 3 and the 5’ extreme of the exon between introns 5 and 6. Homologous sequences of selected samples of *Apiospora* available in public databases (International Nucleotide Sequence Database Collaboration, INSDC, Arita et al. 2021) were included, adding also sequences of *Arthrinium* as outgroup. The sequences employed (Suppl. material 1) were mainly retrieved from Smith et al. (2003), Singh et al. (2012), Crous and Groenewald (2013), Crous et al. (2015, 2020), Senanayake et al. (2015), Dai et al. (2016, 2017), Wang et al. (2017, 2018), Jiang et al. (2018, 2019, 2020), Liu et al. (2019), Pintos et al. (2019), Yan et al. (2019), Yang et al. (2019), Senanayake et al. (2020), Pintos and Alvarado (2021), Kwon et al. (2021), Phukhamsakda et al. (2022) and Samarakoon et al. (2022). Sequences first were aligned in MEGA software with its Clustal W application and then corrected manually. Gblocks (Castresana 2000) was employed to remove 191 ambiguously aligned positions from ITS rDNA, resulting in a final alignment with 188/463/82 (ITS rDNA), 126/786/64 (28S rDNA), 358/847/52 (tef1) and 576/807/47 (tub2) variable sites/ total sites/ sequences.

## ﻿Results

**Figure 1.**
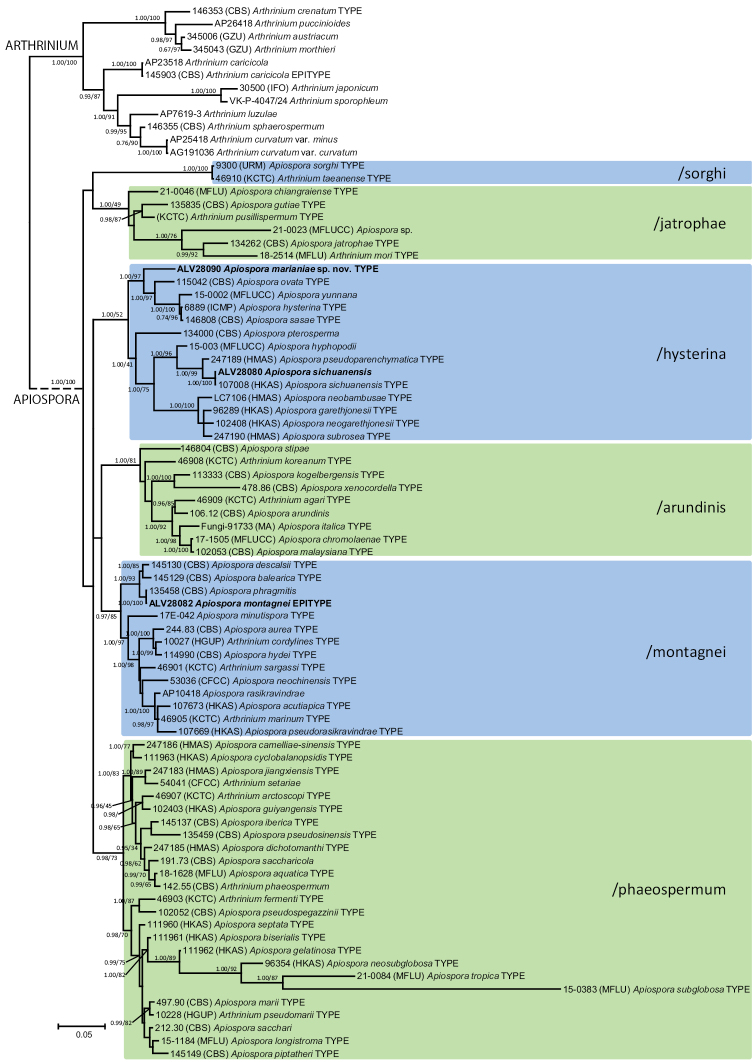
Majority rule consensus (50%) ITS rDNA- 28S rDNA- tef1- tub2 phylogram of the Apiosporaceae obtained in MrBayes from 4 875 sampled trees. Nodes were annotated if supported by > 0.95 Bayesian PP (left) or > 70% ML BP (right).

The phylogenetic analysis of sequenced species of *Apiospora* including ITS1-5.8S-ITS2 and LSU rDNA, as well as exon and intron regions from tef1 and tub2 genes (Fig. 1) resulted in six significantly supported major clades: 1) /sorghi (apparently containing a single species, *Ap.sorghi* (J.D.P. Bezerra, C.M. Gonçalves & C.M. Souza-Motta) X.G. Tian & Tibpromma = *Ar.taeanense* S.L. Kwon, S. Jang & J.J. Kim), 2) /jatrophae, 3) /hysterina, 4) /arundinis, 5) /montagnei, and 6) /phaeospermum. These clades, identified in the present work for the first time, maybe represent monophyletic lineages that could be interpreted as sections or subgenera inside *Apiospora*. However, this hypothesis should be further tested with additional data from less variable DNA regions, since ITS1 rDNA and introns can be easily misaligned.

Among the samples analyzed in the present study (Suppl. material 2), five collections of *Apiospora* were found growing on Arundoaff.micrantha (the supposed host plant of the lectotype of *Sphaeriaapiospora*) in Girona (northeastern Spain) and Mallorca (Balearic Islands, Spain). Four of them were not genetically different from *Ap.phragmitis*, and one matched *Ap.sichuanensis*. Another seven samples found growing on *Arundodonax* in Mallorca matched *Ap.phragmitis*, and one *Ap.sichuanensis*. Due to these results and the data available from Mediterranean species of *Apiospora*, *Ap.phragmitis* is here considered the most probable synonym of *Ap.montagnei*, and an epitype for this species selected among the samples analyzed in the present work. Finally, two samples found on *Phleumpratense* in Mallorca turned out to represent a previously unknown phylogenetic lineage, which is given a new name below.

### ﻿Taxonomy

#### ﻿ Apiospora montagnei

Taxon classificationFungiAmphisphaerialesApiosporaceae

Sacc., Atti Soc. Veneto-Trent. Sci. Nat. 4: 85. 1875.

A97E5C52-49B8-5F73-ADCB-DCA598125898

Fig. 2C–2N

Sphaeria apiospora Durieu & Mont., Expl. Sci. Alg., Fl. Algér. 1, livr. 13: 492. 1849. [replaced name]
Hypopteris apiospora (Durieu & Mont.) Berk., Hooker’s J. Bot. Kew Gard. Misc. 6: 227. 1854.
Arthrinium phragmitis Crous, IMA Fungus 4: 147. 2013.
Apiospora phragmitis (Crous) Pintos & P. Alvarado, Fungal Systematics and Evolution 7: 206. 2021.

##### Sexual morph.

Stromata solitary to gregarious, immersed to erumpent, fusiform, with the long axis broken at the top by one or two cracks, (0.5–)2.1–2.9(–4) × (0.2–)0.25–0.35(–0.5) mm (n = 20). Ascomata uniseriate or irregularly arranged beneath stromata, pseudothecial, black, globose to subglobose with a flattened base, (150–)159–183(–200) µm high × (200–)247–278(–300) µm wide (n = 35), with a conspicuous periphysate ostiole. Peridium composed of 5 or 6 layers of brown to hyaline cells arranged in textura angularis. Hamathecium paraphyses hyphae-like, up to 4 µm wide. Asci broadly cylindrical, clavate, with an indistinct pedicel, rounded at the apex, lacking apical apparatus, (72–)99–111(–115) × (14–)15.5–16.5(–18) µm (n = 25). Ascospores uniseriate or biseriate, clavate to fusiform, straight or slightly curved, with narrowly rounded ends, composed of a large upper cell and a small lower cell, hyaline, smooth-walled, measuring (21–)23–24.5(–25) × (6–)6.3–7.1(–8) µm (n = 30).

**Figure 2.**
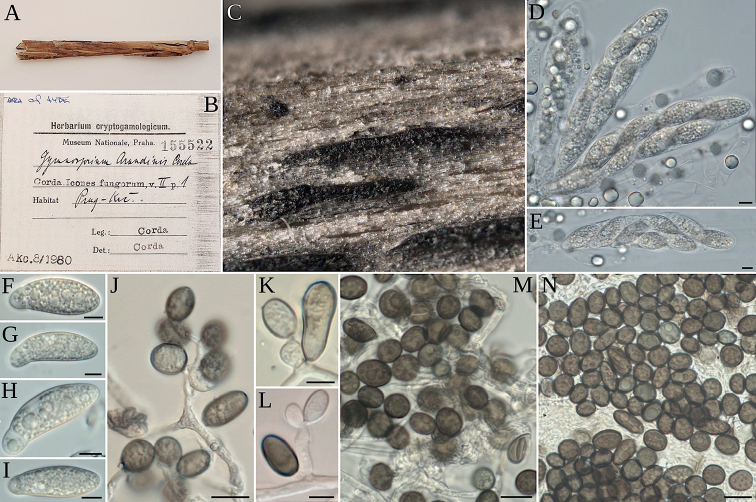
**A, B***Gymnosporiumarundinis* (original material from PRM) **A** substrate of the type **B** label of the original collection of *Gymnosporiumarundinis***C–N***Apiosporamontagnei* (AP301120) **C** stromata on host **D, E** asci and periphyses **F–I** ascospores **J–L** conidiogenous cell with conidia **M–N** conidia in face and side view. Scale bars: 100 µm (**C**); 5 µm (**D–I**); 10 µm (**J–N**).

##### Asexual morph.

Mycelium consisting of hyaline, smooth, branched, septate hyphae 1–4 μm in diam. (n = 20). Conidiophore mother cell from hyaline to brown, solitary or aggregated in groups on hyphae, subsphaerical to lageniform or ampuliform, measuring (4–)6.6–8(–10) × (3–)4.5–5.1(–6) µm (n = 10). Conidiophores cylindrical, straight to flexuous, some of them branched, hyaline, measuring (10–)18–34(–45) × (1.5–)1.6–1.8(–2) µm (n = 20). Conidiogenous cells doliiform to lageniform or ampuliform, hyaline, measuring (10–)11.5–13.1(–15) × (2–)4.3–5.1(–6) µm (n = 20). Conidia ellipsoidal to ovoid, smooth to finely roughened, with an equatorial germ slit of paler pigment, measuring (9–)10.3–11.3(–12) µm in surface view, (5–)6.2–7.2(–8) µm in side view (n = 25). Sterile cells ellipsoidal to clavate, measuring 13–16 µm (n = 25).

##### Culture characteristics.

Colonies flat, spreading, with moderate aerial mycelium. On MEA, surface dirty white with pale rose patches, reverse luteous. Occupying an entire 90 mm Petri dish in 14 days at room temperature, sporulating four weeks after culture.

##### Epitype.

Spain: Catalonia, Girona, L´Escala, on *Arundomicrantha*, 30 November 2020, leg. Marc Grañem, AP301120 (epitype selected here PC:0125164, ex-type culture CBS 148707; iso-epitype WU-MYC0044524, ex-type culture CBS 148708).

##### Other specimens examined.

Spain: Balearic Islands, Mallorca, Esporlas, on *Arundodonax*, 14 December 2020, leg. Ángel Pintos, AP141220 (WU-MYC0044527). Balearic Islands, Mallorca, Palma de Mallorca, Torrente de Establiments, on *Arundomicrantha*, 19 April 2021, leg Ángel Pintos, AP19421 (WU-MYC0044523). Balearic Islands, Mallorca, Puerto de Alcudia, on *Arundodonax*, 2 April 2021, leg. Ángel Pintos, AP2421 (WU-MYC0044522). Balearic Islands, Mallorca, Puerto de Andratx, on *Arundodonax*, 5 April 2021, leg. Ángel Pintos, AP5421 (WU-MYC0044529). Balearic Islands, Mallorca, Puerto de Soller, on *Arundodonax*, 3 April 2021, leg. Ángel Pintos, AP3421 (WU-MYC0044528). Balearic Islands, Mallorca, Puigpunyent, on *Arundomicrantha*, 11 December 2020, leg. Ángel Pintos, AP111220A. Balearic Islands, Mallorca, Soller, on *Arundodonax*, 4 April 2021, leg. Ángel Pintos, AP4421 (WU-MYC0044530). Balearic Islands, Mallorca, Universitat de les Illes Balears (UIB), on *Arundodonax*, 28 December 2020, leg. Ángel Pintos, AP281220 (WU-MYC0044521). Catalonia, Barcelona, Premia de Dalt, on *Arundodonax*, 10 October 2020, leg Miguel Mir, AP101020. Catalonia, Girona, Bescanó, on *Arundomicrantha*, 29 November 2020, leg. Marc Grañem, AP291120 (WU-MYC0044526).

##### Notes.

The phylogenetic boundaries of *Apiospora* were recently discussed by Pintos and Alvarado (2021), who selected a lectotype (PC:0125160) for *S.apiospora*, the basionym of the type species *Ap.montagnei*. In the present study, an epitype (PC:0125164) of *Ap.montagnei* is selected among modern collections growing on the same host in Girona, Spain (about 100 km south of the type locality, Perpignan, France). All samples growing on the same host collected in Girona or the Balearic Islands (Spain) are genetically identical to the epitype, and match the phylogenetic clade formerly known as *Ap.phragmitis*, excepting one that matches *Ap.sichuanensis*, but the ascospores of this species (29–48 × 7–10.5 μm, Samarakoon et al. 2022) clearly exceed those of *Ap.montagnei* (21–25 µm, Pintos and Alvarado 2021). Therefore, on the basis of these results, it is here hypothesized that *Ap.montagnei* is a prioritary synonym of *Ap.phragmitis*.

#### ﻿ Apiospora marianiae

Taxon classificationFungiAmphisphaerialesApiosporaceae

sp. nov. Pintos & P. Alvarado

F964E50A-A431-5CA3-9F35-52FF13F3FC1D

843732

Fig. 3

##### Etymology.

The epithet refers to Marian Mateu, the person who found the holotype collection and beloved wife of the first author.

**Figure 3.**
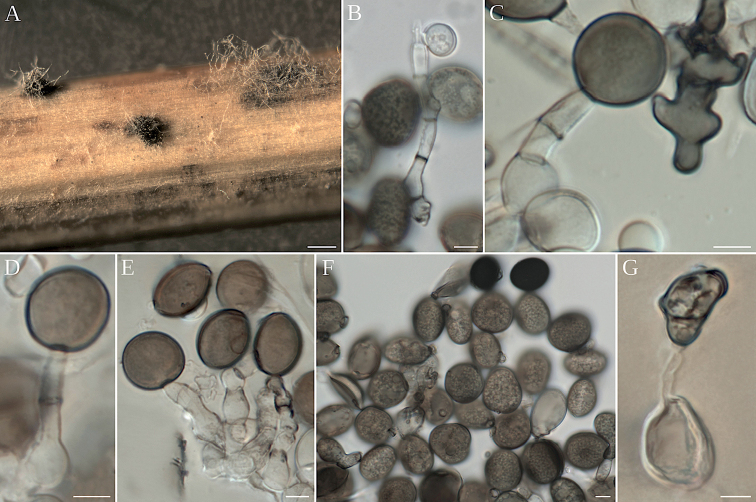
*Apiosporamarianiae* (AP18219) **A** colony on culture **B–E** conidiophore mother cell with septate conidiophore giving rise to conidia, in C irregularly lobate sterile cell **F** conidia in face and side view **G** conidiophore mother cell with irregular conidia from agar. Scale bars: 100 µm (**A**); 5 µm (**B–G**).

##### Holotype.

Spain: Balearic Islands, Palma de Mallorca, on *Phleumpratense*, 18 February 2019, leg. Marian Mateu AP18219 (holotype CBS 148710).

##### Asexual morph.

Mycelium branched, septate, brown to dark brown. Conidiomata sporodochial, punctiform, scattered or confluent, black, (150–)169–203(–220) µm long × (70–)76–88(–100) µm wide (n = 30). Conidiophore mother cells on the surface of the stroma lageniform to ellipsoidal or doliiform, hyaline to brown, measuring (12–)13.4–14.2(–15) × (4–)6–7.2(–8) µm (n = 10). Conidiophores arising from conidiogenous mother cells, basauxic, cylindrical, straight to flexuous, hyaline except the thin transverse septa, smooth, measuring (19–)28–44(–55) × 3–3.6(–4) µm (n = 25). Conidiogenous cells monoblastic, integrated, terminal and intercalary, cylindrical. Conidia brown, solitary; face view: globose to ovate or ellipsoidal, with pale germ slit, (11–)12.1–13.5(–18) µm in diam. (n = 70); side view: lenticular, (8–)8.4–9.2(–10) µm in diam. (n = 30). Sterile cells only seen in culture, brown, granulate, irregularly lobed, (19–)25–31(–35) × (6–)8.15–8.45(–12) µm diam. (n = 40).

##### Culture characteristics.

colonies in MEA white and cottony, with gray patches, reverse gray. Reaching 80–90 mm in diam, in 14 days at room temperature, sporulating after 5 weeks.

##### Other specimens examined.

Spain: Balearic Islands, Palma de Mallorca, Establiments, on *Phleumpratense*, 30 November 2019, leg. Angel Pintos, AP301119.

##### Notes.

According to phylogenetic inference, *Ap.ovata* is the species most closely related to *Ap.marianiae*, but their ITS rDNA sequences are only 94% similar (including gaps). Their conidia are both oval to broadly ellipsoid, but those of *Ap.marianiae* measure 11–15 µm in diam., while those of *Ap.ovata* are longer, measuring about 18–20 µm in diam. in surface view.

#### ﻿ Apiospora sichuanensis

Taxon classificationFungiAmphisphaerialesApiosporaceae

Samarak., Jian K. Liu & K.D. Hyde, in Samarakoon, Hyde, Maharachchikumbura, Stadler, Gareth Jones, Promputtha, Suwannarach, Camporesi, Bulgakov & Liu, Fungal Diversity 112: 21. 2022.

019A4CE9-4125-51B9-957E-600C9B3252A9

Fig. 4

##### Asexual morph.

Mycelium branched, septate, brown. Conidiomata on host parallel to the longitudinal axis of the stem, subepidermal, opening after the dehiscence of the host epidermis, containing a black conidial mass, measuring (400–)600–950(–1000) × (275–)300–550(–600) µm (n = 40). Conidiophore mother cells arising from the stroma, lageniform to ampuliform, pale brown, with superficial granular depositions, (5–)6–10(–16) × (3–)5–7(–8) µm (n = 30). Conidiophores basauxic, cylindrical, straight or flexuous, sometimes with a thin septum, hyaline to brown, smooth, with granular pigments, (20–)43–67(–80) µm in length × (2–)2.2–3.4(–4) µm wide (n = 50). Conidia globose, subcylindrical to ovate, polygonal or obpyriform, with a lateral germ slit over the entire length, brown, smooth, irregularly lobed, measuring (10–)23–31(–35) × (5–)9–13(–14) µm (n = 30).

**Figure 4.**
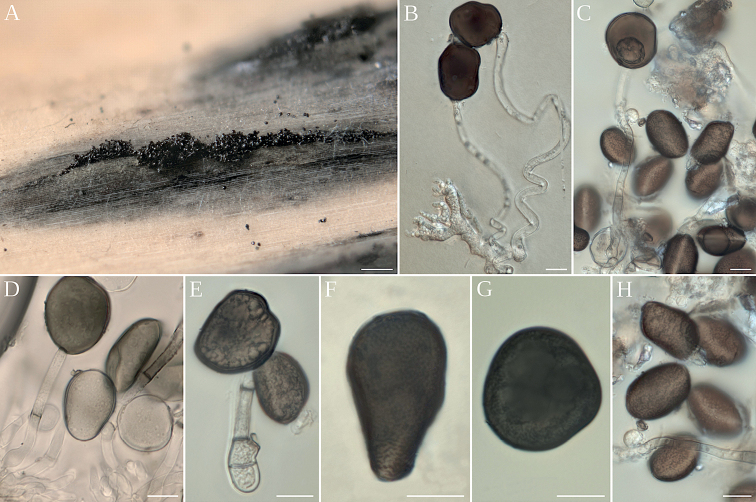
*Apiosporasichuanensis* (AP121220) **A** conidiomata on host **B** germinated conidia **C–E** conidiophore mother cell giving rise to septate conidiophore with conidia **F–H** conidia. Scale bars 100 µm (**A**); 10 µm (**B–H**).

##### Culture characteristics.

colonies on MEA 70–90 mm in diam. after 14 days at room temperature, flat, spreading, first white and cottony, later becoming gray, reverse dark gray. On PDA (200 g/L potato, 20 g/L dextrose, 20 g/L agar, pH 7.0), 80–90 mm in diam, after 14 days at room temperature, sporulating after 4–5 weeks, white cottony at first, then becoming gray with luteous patches, reverse dark gray.

##### Specimens examined.

Spain: Balearic Islands, Mallorca, Palma de Mallorca, Torrente de Soller, on *Arundodonax*, 12 December 2020, leg. Ángel Pintos, AP121220 (WU-MYC0044525). Catalonia, Girona, Bescanó, on *Arundomicrantha*, 15 November 2020, leg. Marc Grañem. AP151120.

##### Notes.

*Ap.sichuanensis* is genetically close to *Ap.pseudoparenchymatica* (M. Wang & L. Cai) Pintos & P. Alvarado, but the fruiting body of the former is an acervulus and that of the latter a sporodochium. In addition, the conidia of *Ap.sichuanensis* are 10–35 × 5–14 µm, longer and narrower than those of *Ap.pseudoparenchymatica*, which measure 13.5–27.0 × 12.0–23.5.

## ﻿Discussion

In the present work, several samples of *Apiospora* growing on Arundoaff.micrantha (the most probable host plant of the lectotype collection of *S.apiospora*, PC:0125160, Pintos and Alvarado 2021) were analyzed in order to clarify the identity of the type species of *Apiospora*, *Ap.montagnei* (≡ *Sphaeriaapiospora*). In previous works (Crous and Groenewald 2013, Pintos et al. 2019), four species of *Apiospora* were found on *Arundo* spp.: *Ap.iberica*, *Ap.italica*, *Ap.marii*, and *Ap.phragmitis*. In the present work, the first record of *Ap.sichuanensis* in Europe was found growing also on *Arundo* spp. However, the ascospore dimensions reported in the protologues of *Ap.iberica* (29–34 × 6–8 μm, Pintos et al. 2019) and *Ap.sichuanensis* (29–48 × 7–10.5 μm, Samarakoon et al. 2022), clearly exceed those measured on the lectotype of *S.apiospora* (PC 0125160, 21–25 µm, Pintos and Alvarado 2021), and other original material of this species (23–28 µm, Hyde et al. 1998). On the contrary, ascospores of *Ap.italica*, *Ap.marii* and *Ap.phragmitis* are not significantly different from those of the lectotype of *S.apiospora*.

All samples of *Apiospora* found on Arundoaff.micrantha with an ascospore size matching that of *Ap.montagnei* are genetically identical to *Ap.phragmitis*. A single collection of *Ap.italica* (MA-Fungi 91733, Pintos et al. 2019), and another one of *Ap.marii* (MA-Fungi 91735, Pintos et al. 2019) were previously found on *Arundodonax*, a host plant were *Ap.phragmitis* and *Ap.sichuanensis* can occur too. Despite the lack of collections confirming it, it is certainly possible that *Ap.italica* and *Ap.marii* grow also on Arundoaff.micrantha, as these species have been found also on other host plants, especially *Phragmites*, but also *Ampelodesmos* and many others (*Arundinaria*, *Beta*, oats, seaweeds). *Apiosporamarii* has been found in southern, central and northern Europe (Spain, Italy, Austria, The Netherlands, Sweden) and Asia (China, Korea), being most probably a widespread species. By way of contrast, *Ap.italica* and *Ap.phragmitis* have been found only in the Mediterranean region.

Given the wide host plant range observed in *Apiospora*, other species which have not been found yet on *Arundo* could be collected on this host plant genus in the future, reducing the reliability of this character for diagnosis. Of those species occurring in the Mediterranean region, some present ascosopores differing in size from *Ap.montagnei* (i.e., *Ap.balearica* (Pintos & P. Alvarado) Pintos & P. Alvarado, *Ap.hysterina* (Sacc.) Pintos & P. Alvarado). Others, such as *Ap.descalsii* (Pintos & P. Alvarado) Pintos & P. Alvarado, are apparently rare, and the probability of a synonymy with *Ap.montagnei* is therefore low. The sexual state of *Ap.rasikravindrae* (Shiv M. Singh, L.S. Yadav, P.N. Singh, Rah. Sharma & S.K. Singh) Pintos & P. Alvarado produces ascospores measuring 21.5–24.5 × 7–9.5 µm (Dai et al. 2017), therefore fitting the size range observed in *S.apiospora* lectotype, but the synonymy is here rejected because this species has never been found yet on *Arundo* sp. (only known to grow on ornamental *Phyllostachys* and bamboo plants in Mallorca). Unfortunately, the sexual state of multiple species occurring in the Mediterranean region (i.e., *Ap.aurea* (Calvo & Guarro) Pintos & P. Alvarado, *Ap.esporlensis* (Pintos & P. Alvarado) Pintos & P. Alvarado, *Ap.hispanica* (Larrondo & Calvo) Pintos & P. Alvarado, *Ap.mediterranea* (Larrondo & Calvo) Pintos & P. Alvarado, *Ap.piptatheri* (Pintos & P. Alvarado) Pintos & P. Alvarado, *Ap.serenensis* (Larrondo & Calvo) Pintos & P. Alvarado, as well as the new species introduced in the present work, *Ap.marianiae*) is still unknown, and therefore they cannot be compared yet with the lectotype of *S.apiospora* (≡ *Ap.montagnei*). However, these seem to be rare species, and they have never been found on *Arundo* yet, so the synonymy is here considered much less probable.

A classical candidate synonym of *Ap.montagnei*, *Ap.arundinis* (Corda) Pintos & P. Alvarado (Crous and Groenewald 2013), has not been found yet in the western Mediterranean region (Pintos et al. 2019, Pintos and Alvarado 2021), but it seems to be widespread elsewhere, occurring in temperate, cold and also subtropical countries (Crous and Groenewald 2013). In Spain, it has been found in ornamental *Bambusa* plants in Galicia (north-western Spain), but not in the Mediterranean border with France (closer and ecologically more similar to Perpignan, the type locality of *Ap.montagnei*). Sequenced samples of *Ap.arundinis* found growing in *Arundo* are currently lacking, but the type collection of its basionym, *Gymnosporiumarundinis* Corda was reported to grow on reeds and grasses near Prague by Corda (1838). An original sample kindly loaned by the Prague herbarium (PRM 155522) was found to present globose conidia 5–7 µm in diam., a size compatible with that observed in the clade identified as *Ar.arundinis* by Crous and Groenewald (2013), but also others, such as *Ap.descalsii*, *Ap.italicum*, *Ap.jiangxiensis* (M. Wang & L. Cai) Pintos & P. Alvarado, *Ap.malaysiana* (Crous) Pintos & P. Alvarado, *Ap.pseudospegazzinii* (Crous) Pintos & Alvarado or *Ap.sacchari* (Speg.) Pintos & P. Alvarado. Interestingly, the host plant of this original collection of *G.arundinis* loaned by PRM was not *Arundo*, but very probably *Phalarisarundinacea*. The identity of *Ap.arundinis* needs to be further investigated, and an epitype from Prague selected, to ascertain if the name is being correctly applied.

Therefore, on the basis of the data currently available (host plants, ascospore sizes, abundances, distributions), it is here hypothesized that the lectotype of *S.apiospora* (≡ *Ap.montagnei*) is not genetically different from the clade of *Ap.phragmitis*. An epitype of *S.apiospora* (≡ *Ap.montagnei*) from Girona (Spain, about 100 km south of Perpignan, the locality where the lectotype was found) is here chosen, and a synonymy between *Ap.montagnei* and *Ap.phragmitis* is suggested.

## Supplementary Material

XML Treatment for Apiospora montagnei

XML Treatment for Apiospora marianiae

XML Treatment for Apiospora sichuanensis

## Supplementary materials

Supplementary material 1 Table S1 Data type Sequences. Explanation note Sequences produced in the present work (in bold) and retrieved from databases. https://binary.pensoft.net/file/734070This dataset is made available under the Open Database License (http://opendatacommons.org/licenses/odbl/1.0/). The Open Database License (ODbL) is a license agreement intended to allow users to freely share, modify, and use this Dataset while maintaining this same freedom for others, provided that the original source and author(s) are credited.Ángel Pintos, Pablo Alvarado

Supplementary material 2 Table S2 Data type Sequences. Explanation note Samples analyzed in the present work. https://binary.pensoft.net/file/734071This dataset is made available under the Open Database License (http://opendatacommons.org/licenses/odbl/1.0/). The Open Database License (ODbL) is a license agreement intended to allow users to freely share, modify, and use this Dataset while maintaining this same freedom for others, provided that the original source and author(s) are credited.Ángel Pintos, Pablo Alvarado
