# ﻿Corrigendum: Pintos Á, Alvarado P (2022) New studies on *Apiospora* (Amphisphaeriales, Apiosporaceae): epitypification of *Sphaeriaapiospora*, proposal of *Ap.marianiae* sp. nov. and description of the asexual morph of *Ap.sichuanensis*. MycoKeys 92: 63–78. https://doi.org/10.3897/mycokeys.92.87593

**DOI:** 10.3897/mycokeys.92.94104

**Published:** 2022-09-13

**Authors:** Ángel Pintos, Pablo Alvarado

**Affiliations:** 1 Interdisciplinary Ecology Group, Universitat de les Illes Balears, Palma de Mallorca, Spain Universitat de les Illes Balears Palma de Mallorca Spain; 2 ALVALAB, Oviedo, Spain ALVALAB Oviedo Spain

In the published version of the article “New studies on *Apiospora* (Amphisphaeriales, Apiosporaceae): epitypification of *Sphaeriaapiospora*, proposal of *Ap.marianiae* sp. nov. and description of the asexual morph of *Ap.sichuanensis*” by Pintos & Alvarado, MycoKeys 92: 63–78 (2022) the culture designated as holotype of *Apiosporamarianiae* was not indicated as being preserved in a metabolically inactive state. As a consequence, the proposed new species name is invalid due to Art. 40.8 of the Shenzhen Code (Turland et al. 2018).

**The species is validated herein**:

*Apiosporamarianiae* Pintos & P. Alvarado, sp. nov. MB 845455 For a detailed description see: Pintos & Alvarado, MycoKeys 92: 70 (2022). Holotype: CBS 148710 (preserved in a metabolically inactive state).

In addition, the epitypification of *Sphaeriaapiospora* was published without an identifier and is therefore not Code compliant (Art. F.5.4, Shenzhen Code).

**Here, we designate the epitype again**:

*Apiosporamontagnei* Sacc., Nuovo Giorn. Bot. Ital. 7: 306 (1875). Replaced synonym: *Sphaeriaapiospora* Durieu & Mont., Exploration scientifique de l’Algérie 1(13): 482, tab. 25, fig. 1 (1849)

Supported lectotype: PC 0125160.

Epitype (designated here, MBT 10008889): Spain, Catalonia, Girona, L’Escala, 42°07’06.5”N 3°09’01.0”E, on dead culms of *Arundomicrantha*, 30 Nov. 2020, Marc Grañem, PC 0125164; exepitype CBS 148707.
